# Rapid Collection of Biospecimens by Automated Identification of Patients Eligible for Pharmacoepigenetic Studies

**DOI:** 10.3390/jpm3040263

**Published:** 2013-09-26

**Authors:** Yan V. Sun, Robert L. Davis

**Affiliations:** 1 Department of Epidemiology, Rollins School of Public Health, Emory University, Atlanta, GA 30322, USA; 2 Department of Biomedical Informatics, School of Medicine, Emory University, Atlanta, GA 30322, USA; 3 Center for Health Research, Kaiser Permanente Georgia, Atlanta, GA 30305, USA; E-Mail: robert.l.davis@kp.org

**Keywords:** electronic medical record, epigenetics, DNA methylation, epigenome, pharmacogenomics, pharmacogenetics, pharmacoepigenomics, epigenetic epidemiology

## Abstract

Epigenetics plays an important role in regulating gene expression, and can be modified by environmental factors and physiological conditions. Studying epigenetics is a promising approach to potentially improving the diagnosis, prevention and treatment of human diseases, and to providing personalized medical care. However, the role of epigenetics in the development of diseases is not clear because epigenetic markers may be both mediators and outcomes of human diseases. It is particularly complicated to study pharmacoepigenetics, as medication use may modify the epigenetic profile. To address the challenges facing pharmacoepigenetic research of human diseases, we developed a novel design to rapidly identify, contact, and recruit participants and collect specimens for longitudinal studies of pharmacoepigenetics. Using data in real-time from electronic medical record systems, we can identify patients recently start on new medications and who also have a blood test. Prior to disposal of the leftover blood by the clinical laboratory, we are able to contact and recruit these patients, enabling us to use both their leftover baseline blood sample as well as leftover specimens at future tests. With treatment-naïve and follow-up specimens, this system is able to study both epigenetic markers associated with disease without treatment effect as well as treatment-related epigenetic changes.

## 1. Introduction

### 1.1. Epigenetics and Human Diseases

Epigenetic markers can be modified by environmental exposures [1], and are partially determined by genetic variants [2]. Epigenetic modification, through DNA methylation (DNAm) and other molecular mechanisms, can regulate gene expression levels and may be an important molecular mechanism underlying disease development. Epigenetics provides molecular adaptability [3,4,5] and complexity [6] in the human genome by allowing gene expression to respond to environmental changes. The epigenetic modification can be inherited across cell generations to have a long-term impact on the development of chronic diseases. DNAm is an essential epigenetic mechanism for normal development and is associated with several key processes linked to chronic disease. 

Reports have suggested that epigenetic alteration is linked to the development of chronic inflammation [7,8], which is related to the pathophysiological processes of multiple chronic diseases. Further, epigenetic marks can be modified by many risk factors (e.g., age [9,10], nutrition [11], and other environmental factors) of chronic diseases, and may play a role in mediating the molecular effects of these risk factors. 

### 1.2. Pharmacoepigenetic Studies

Pharmacoepigenetics (PEGx) is regarded as the study of epigenetic modifications, which may in turn affect or predict individual responses to therapies in terms of efficacy and adverse effects. Thus, the epigenetic profile may serve as an important biomarker representing the physiological responsiveness to the treatment, and may play a critical role in mediating the drug effect. Although the most promising PEGx studies have been demonstrated in the treatment of cancer, PEGx studies of therapies for other diseases may lead to a better understanding of inter-individual differences in drug response. In the past decade, researchers have investigated the epigenetic regulation of ADME genes (genes related to drug absorption, distribution, metabolism, and excretion), which play a critical role in the biological response to the drug targets. Ivanov *et al*. recently reviewed the PEGx research of ADME genes and other candidate genes [12], which are potential targets of epigenetic regulation. With the advancement of genomic technologies, such as the microarray-based and high-throughput sequencing-based methods, studying epigenome-wide markers of drug response in large populations is feasible. Epigenetic profiles have great potential to be used as biomarkers for predicting the responses to drugs, and may eventually assist with identifying personalized therapies for many different diseases.

### 1.3. Epigenetic Modification by Intervention

Unlike the fixed genetic profile of an individual, epigenetic profiles can be modified by age [13] and environmental factors, such as smoking [14,15,16], pesticides [17] and toxicant exposures [18]. A change in an individual’s physiological condition (e.g., developing a chronic diseases) may modify epigenetic markers, while an intervention (e.g., cessation of cigarette smoking; dispensation of a new medication) may also change the epigenetic profile over a relatively short period of time. Because the disease-associated epigenetic markers can be the cause, consequence or confounder of disease, the timing of the epigenetic measurements in the study of personalized medicine is critical to capture the right epigenetic state, and to fully understand the relationship between epigenetic profiles and medication use.

### 1.4. Pharmacogenetic (PGx) and PEGx Research Using Electronic Medical Record (EMR)

EMR systems are able to capture and integrate data on most aspects of clinical care over time, with the data being represented in the form of standardized codes, controlled vocabularies and text-based clinical narratives. Nationwide initiatives in the US and European countries have promoted electronic data storage and sharing in health care systems to improve clinical care and decision-making. The availability of rich clinical phenotypes from the EMR databases has enabled genetic association studies using genome-wide association (GWA) designs [19,20]. These studies can efficiently identify target samples with DNA and phenotype of interest from EMR, and have successfully identified genetic loci associated with disease traits [21,22], particularly with those clinical outcomes related to pharmaceutical treatments in PGx research [23]. Detailed longitudinal profiles based on clinical and pharmacy data can be extracted from EMR databases to investigate the genetic associations with treatment outcomes, such as drug efficacy and adverse effect. These tangential phenotype and medication information could facilitate secondary analyses within the targeted study, such as correction for confounders, and assessment of interaction effects. In addition, existing blood tests could also be used to identify additional phenotypes to evaluate the PGx study of effectiveness and adverse effects. This approach can also be applied to study the genetic determinants of severe adverse effects, since the cases can be more effectively identified and recruited using the large EMR system, which typically has hundreds of thousands of participants, if not millions. 

Because epigenetic modifications can regulate gene expression levels, they may also play an important role in the molecular mechanism underlying the pathophysiology of the human diseases, as well as in the responsiveness and adverse effect of a certain medication. The epigenetic state of an individual may capture the cumulative environmental exposures in early life, and may serve as a biomarker of physiological plasticity in response to the medication. However, there are several major challenges facing epigenetic research in human populations [24,25] centered around the timing of the sample collection. In this paper, we describe our efforts in addressing two timing-related issues: the potential influences of medication on the epigenetic profile, and the background longitudinal changes of the epigenome irrespective of medication exposure. We present here a design to rapidly identify informative cases and collect biospecimens to capture the time-sensitive epigenetic profile for future studies of PEGx. 

## 2. A Novel Design of PEGx Using EMR Database

In a typical PGx study, participants are usually required to be free of medication for a period of time (so-called “washout period”) before the trial starts. It is usually scheduled prior to initiation of the treatment and/or the placebo arms. This “washout period” is necessary to remove the residual effects of any previous medications to assess the actual change of clinical phenotypes for a given medication. The US Food and Drug Administration (USFDA) recommends to keep a minimal washout period of 5 half lives of the drug. In practice, many studies choose a sufficiently long period (e.g., 14 days) for consistency and convenience between treatment arms and phases. Although this might be a valid approach to studying epigenetic predictors of drug response, such designs can be problematic if there are concerns about potential risks of disease progression during the washout period. In addition, clinical trials are typically associated with very high costs, which can limit the sample size for detecting the epigenetic association. Perhaps most importantly, though, is that the washout period may not be sufficient to recover the epigenetic changes caused by the medication. Thus, the epigenetic profile measured on the samples collected after a washout period can still remain partially modified by the medication, complicating interpretation of the results. To minimize the treatment effect on epigenetics, to reduce the potential risk due to the washout period, and to improve the efficiency of the sample collection for the PEGx research, we developed a novel approach for rapidly collecting leftover blood samples from routine laboratory tests by identifying and recruiting qualified patients using an automated search in the electronic medical record (EMR) system. 

**Figure 1:**
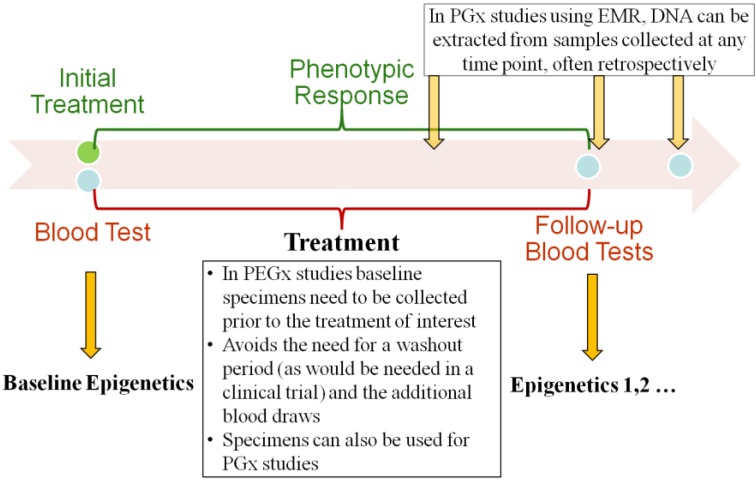
Longitudinal blood collection for pharmacoepigenetics (PEGx) studies.

**Figure 2:**
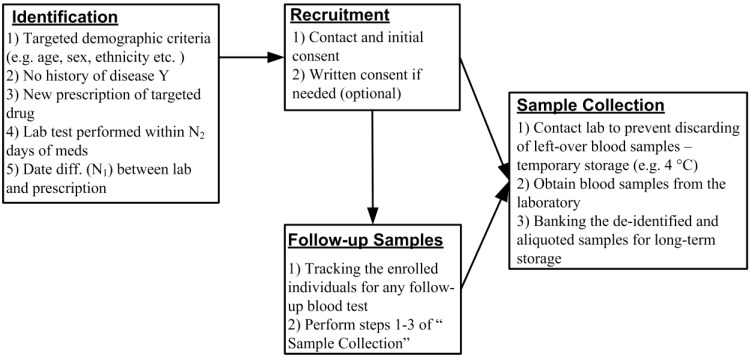
Workflow of the rapid collection of biospecimen.

We have developed a novel recruitment method that uses a real-time EMR database harboring the clinical, laboratory, demographic and pharmacy data. We propose the elimination of the washout period by identifying and recruiting treatment-naïve patients who are starting a targeted medication. At the same time point, we are able to collect leftover blood samples used for the routine laboratory test for PEGx research (Figure 1). The phenotypic responses to the treatment can be obtained from the EMR database. Here we describe the steps by which it would be possible to undertake this study design strategy for future PEGx studies. The detailed procures are outlined in Figure 2, and described in the following sections.

### 2.1. Rapid Identification and Recruitment of Treatment-Naïve Participants

We use four key criteria to identify informative phenotypes based on EMR data in addition to typical inclusion criteria such as age, ethnicity and disease diagnosis. First, the participant must have a ***new prescription*** of the targeted medication (e.g., drug X). In other words, the identified prescription of drug X is the only observation of that specific drug for that specific person in the EMR dataset. Second, the participant must have a ***qualified blood test*** performed within the past N_1_ (e.g., five) days, so as to allow the collection of leftover blood. It is important to note here that the clinical lab policies typically require that all lab samples be retained for seven days following a blood draw, thus allowing time for identification of new qualified patients (from Step 1) who have recently had a blood draw and have an available sample (from Step 2). Third, we require that the time between the prescription of drug X and the recent blood test is ***less than N_2_*** (e.g., three) days. Since blood samples are usually collected right before the medication dispensings, and the epigenetic modification may take weeks to be modified, we use these baseline blood samples to measure the pre-medication epigenetics profile. Forth, we exclude individuals with ***previous diagnosis*** of disease Y, which drug X is used to treat. This restriction of disease Y-free status can exclude the potential modification of the epigenetic profile due to other treatment of disease Y. 

In practice, once qualified individuals are identified, we contact them via phone to obtain initial oral consent allowing us to keep their leftover blood specimens in the laboratory. We then follow up with a written consent through mail that allows for long-term storage of both the initial and specific (or all, depending on the study) follow-up blood samples. During the phone call for the oral consent, we verify the key inclusion and exclusion criteria of the study. This is a critical step, as we have found that the disease-free status inferred from the EMR query may not always be definitive, because some new health plan members may, in fact, not have initiated care yet although they have a history of past disease. Therefore, having the disease or condition confirmed during the initial phone interview can help to identify the truly qualified participants. 

### 2.2. Baseline and Longitudinal Collection of Specimen

The Current Procedural Terminology (CPT) code is maintained by American Medical Association (AMA) to describe medical, surgical, and diagnostic services. The CPT code is designed to communicate uniformly about medical services and procedures among healthcare providers, patients and payers for administrative, financial, and analytical purposes. Similar to ICD-9 or ICD-10 coding for diagnosis, CPT coding identifies the provided services. The five-digit CPT codes between 80001 to 89999 are reserved for pathology and laboratory procedures. To collect specimens for DNA extraction, we focus on laboratory procedures using the blood samples (examples of CPT codes and descriptions were listed in Table 1). 

**Table 1:** Commonly performed laboratory tests using blood samples.

CPT Code	Description (from AMA coding online)
80061	Lipid panel. This panel must include the following: Cholesterol, serum, total (82465), Lipoprotein, direct measurement, high density cholesterol (HDL cholesterol) (83718), Triglycerides (84478)
82040	Albumin; serum, plasma or whole blood
82465	Cholesterol, serum or whole blood, total
80048	Basic metabolic panel (Calcium, total) This panel must include the following: Calcium, total (82310), Carbon dioxide (82374), Chloride (82435), Creatinine (82565), Glucose (82947), Potassium (84132), Sodium (84295), Urea nitrogen (BUN) (84520)
80053	Comprehensive metabolic panel. This panel must include the following: Albumin (82040), Bilirubin, total (82247), Calcium, total (82310), Carbon dioxide (bicarbonate) (82374), Chloride (82435), Creatinine (82565), Glucose (82947), Phosphatase, alkaline (84075), Potassium (84132), Protein, total (84155), Sodium (84295), Transferase, alanine amino (ALT) (SGPT) (84460), Transferase, aspartate amino (AST) (SGOT) (84450), Urea nitrogen (BUN) (84520)
83036	Hemoglobin; glycosylated (A1C)
85025	Blood count; complete (CBC), automated (Hgb, Hct, RBC, WBC and platelet count) and automated differential WBC count

We have found that lipid panels (80061) and basic metabolic panels (80048) are among the most commonly performed laboratory tests using blood samples, and are frequently obtained at the time that a new medication is initiated for a patient. After being used for the prescribed blood test and before being discarded, the leftover blood samples are stored in the laboratory for up to seven days (for quality assurance and control, in case the test needs to be performed again). Assuming that the patients can be identified and contacted quickly after the test, and that the sample is stored under suitable conditions, the blood cells from these “leftover samples” are appropriate material for genetic or epigenetic research. Since these blood tests are routinely ordered and performed to assess the patients’ physiological condition, we can obtain the follow-up blood samples after the initial use of medication with the proper consent from the participants (Figure 2). As part of our design, we can query the EMR database frequently (e.g., twice a week) for any additional blood tests from all consented participants, and contact the laboratory for the newly available follow-up samples. Such a query can be automated to return routine results as part of the biomedical informatics pipeline. The investigators and the interviewers may receive notifications when a qualified participant is identified. These follow-up samples will be linked to the original sample by study identifiers, and banked for the longitudinal study of PEGx. Combining the longitudinal measurements of the epigenetic profile with the matching clinical measurements, we can examine not only the epigenetic association with the medication response and adverse effects, but also the epigenetic change modified by the medication. The intra- and inter-individual difference of the epigenetic profile in response to the medication is a particularly important question in the PEGx study.

## 3. Discussion

PEGx is an emerging field. To our knowledge, well designed population studies of PEGx, particularly the epigenome-wide studies, are very few in numbers. In addition, using convenient samples from a cross-sectional design without taking into account of the treatment effect (*i.e.*, reverse causation) or studying the longitudinal profile, results in inconclusive inference about the association between the epigenetic profile and the pharmacological outcomes. Our strategy of PEGx study using the EMR for sample identification, recruitment and blood collection has a number of advantages as described above. The applicability of this design in any particular EMR system, however, may be limited by the information captured and organized by the system. The strategy as described here requires frequent and routine monitoring of the EMR database to identify qualified participants, and requires quick action to obtain “opt-in” consent from the participants to bank the leftover specimen from the laboratory. Even with the automation of the database queries, a collaborative team needs to be assembled that is able to verify inclusion criteria, recruit participants, contact the laboratory, and to identify and bank relevant specimens. The requirement of the blood test in our design is not necessarily a limitation, because most disease diagnosis and new prescription of medication require some forms of blood test. The researchers can customize their algorithm to target the relevant blood tests accordingly to collect the specimen. 

While a more efficient approach to collecting biospecimens for PEGx study would be to establish a biobank with longitudinal samples, such an effort would require considerable more resources and funding than the approach we outlined here. If all leftover blood samples from participants are banked regardless of their medication use and the status of disease, the researchers may later conduct *in silico* sampling for a PEGx study of a specific medication. However, this approach needs to be regulated by the institutional review board (IRB) for potential ethical concerns. 

Although we present a general design of the PEGx study using EMR systems, researchers may want to pay special attention to the following issues when considering this approach. First, certain drugs have multiple indications beyond the primary disorder to treat. Because this may not be a common concern of most drugs of interest, the current algorithm does not directly address this issue. However, depending on the drug-disease pair of interest, the researchers should consider such drugs based on the domain knowledge, and exclude their influences on a specific PEGx project. Second, our design targets DNAm as a primary epigenetic mark in a population study. DNAm can be measured using genomic DNA extracted from leftover blood samples collected under the laboratory condition. However, we recognize that the stability of other types of epigenetic modification, such as histone modification and micro-RNA is not fully examined for the leftover blood. Further investigation of using leftover blood for other types of epigenetic marks is warranted. Third, a number of environmental factors are known modifiers of epigenetic profile. Measuring all, or even most of them, in single epidemiological study is a very challenging task. These environmental factors cannot be fully controlled for in a PEGx study. The recently proposed “exposome” research may greatly benefit the studies of epigenetic epidemiology, including PEGx. The exposome is a measure of the effects of life-long environmental exposures on health, and addresses the high-dimensional measure of environmental exposures. Certain -omics technologies, such as metabolomics, can measure thousands of chemicals as biomarkers of environmental exposures. With the availability of biospecimens (e.g., serum), we are able to measure them quantitatively (e.g., cotinine for cigarette smoking), and adjust for them to study the epigenetic association with medications. Last, the current pipeline is built based on the US system of EMR, including the clinical and pharmacy databases, as well as the CPT codes for the blood test. For the non-US system, our design is likely to be transferable. However, a thorough comparison is recommended to make necessary modifications of the US-based pipeline. 

Recent studies of chronic obstructive pulmonary disease (COPD) and rheumatoid arthritis (RA), which are related to local and systemic inflammation, identified and replicated a number of DNAm sites associated with disease outcomes using peripheral blood leukocytes (PBLs) [26,27]. The bioinformatic analysis of the gene-specific DNAm sites associated with COPD revealed significant enrichment of immune and inflammatory system pathways, responses to stress and external stimuli, as well as wound-healing and coagulation cascades [26]. These studies strongly supported the epigenetic mechanism related to chronic disease through inflammation, and demonstrated that investigating DNAm in PBLs can lead to discovery of novel genes and pathways associated with chronic conditions. Therefore, studying the PEGx of anti-inflammatory medication such as statins can particularly benefit from this novel study design to understand the bidirectional relationship between the epigenetics and the treatment, as well as the association between epigenetics and human diseases. Previous reports demonstrated that 5-aza-cytidine (5-azaC) dose intensification increased 5-azaC) antineoplastic activity in an animal study [28], and epigenetic variants were associated with the clofarabine-mediated cytotoxicity in lymphoblastoid cell lines [29]. For future PEGx studies, especially for medications functioning through inflammatory pathways and immune systems, our design of collecting longitudinal blood samples before and after the initial treatment can address several challenges facing population studies of epigenetics and epigenomics. Combining epigenetic data with the longitudinal data from the EMR database including the medication dispense data, we are able to study the epigenetic predictors of response to medications, while considering age and other environmental factors, which may also modify epigenetic profile. 

Epigenetic profiles are tissue and cell type-specific [30,31]. Our current design is limited to using PBLs from routine laboratory tests. The epigenetic profile of PBLs can act as a surrogate biomarker for environmental exposures (e.g., parental environment, early-life exposure and nutrition), genetic factors and physiological condition (e.g., biological aging and gender). Thus it may have increased power for detecting the epigenetic association with drug response. In addition to studying the epigenetics as a biomarker of exposure, the choice of PBL is also meaningful to study certain diseases and therapies such as chronic conditions involving the circulation and immunological function. However, since PBL comprise a mixture of cell types and each cell type has the unique epigenetic signature [32], it is possible that the epigenetic associations are confounded by the shift of the proportions of PBL subtypes. We can apply the method developed by Houseman *et al.* [33] to project the subtype proportions of each samples using the methylomic data [16]. Then we can examine the epigenetic association by adjusting for these subtypes of leukocytes including granulocyte, monocyte, natural killer cells (NK), B cell, CD4+ and CD8+ T cells, to address the concern of potential confounding effect. Ideally, using the sorted cells can fully address the heterogeneity of cell proportions of PBLs across individuals. The current technologies of measuring the epigenome (e.g., Illumina Infinium HumanMethylation arrays and next-generation sequencing-based methods) require a large amount of genomic DNA, usually in micro grams. Sorting sufficient amount of homogeneous cells for many PBL subtypes is still not feasible due to the limited amount of cells. However, the improvement of technology may soon allow the high-throughput sequencing using a much smaller amount of DNA, even from a single cell, to ultimately address this issue of heterogeneity. 

Although the design and approach described here focuses on PEGx study, this novel design can be applied to other studies of genetics and epigenetics. For example, this approach can be used in PGx studies that wish to identify informative patients who are initiating a specific medication, and would enable the collection of blood samples without extra effort on the part of the clinical team. This approach can reduce any additional risks for participants in clinical trials that otherwise might arise due to the need for drug-washout period. This design can also be used to study epigenetic changes related to other interventions, which may impact health outcomes via epigenetic mechanisms, such as radiation and chemotherapy for cancer. Both the baseline and the change of the epigenetic profile may predict the effectiveness and the potential side-effect of the treatment, thus providing insights in developing the individualized care to the patients. Finally, this strategy can be used to collect leftover biospecimens (e.g*.*, whole blood, plasma and urine) to study metabolomic, proteomic and other -omic markers for pharmacological outcomes. Further understanding of the relationship between epigenetic variants and clinical outcomes from PEGx research has the potential to contribute to more effective, individualized approaches to evaluation, intervention, and prevention of human diseases, thereby, reduction of disability, death, and mounting health care costs.
